# Supplementary material for: Global scale analysis on the extent of river channel belts
Source: Nat Commun. 2023 Apr 15;14:2163. doi: 10.1038/s41467-023-37852-8 (PMC10105755; doi:10.1038/s41467-023-37852-8)
Supplement: Supplementary file 1 — Supplementary Info [file 41467_2023_37852_MOESM1_ESM.pdf]

Supplementary Material:
Global Scale Analysis on the Extent of River Channel Belts

Björn Nyberg[1,2*], Gijs Henstra[3], Rob Gawthorpe[1], Rodmar Ravnås[3], Juha Ahokas[3]

Department of Earth Sciences, University of Bergen, Allegaten 41, 5020, Bergen, Norway.[1]
Bjerknes Centre for Climate Research, Allegaten 70, 5020, Bergen, Norway.[2]
AkerBP ASA, Oksenøyveien 10, 1366 Lysaker, Norway.[3]

Inventory of Supporting Information

Supplementary Table 1

Supplementary Figure 1

Supplementary Figure 2

Supplementary Figure 3

Supplementary Figure 4

Supplementary Figures and Tables

Supplementary Table 1 Riverine and Lacustrine Distribution – The surface area contribution of each environment in km^2 subdivided by continent. Percentages show the proportion of the total within each category classified as single-threaded. The values are based on the 50% confidence interval of the Global Channel Belt (GCB) map predicting a total channel belt extent of 30.5 x 10^5 km^2 (16.1 to 49.6 x 105 km^2 at a +/- 25% confidence).

	Abandoned Channel Belt	Active Channel Belt	River	Smaller Lakes or Rivers	Channel Belt Total	Lakes	Total
**Africa**	2.88x10^5 (86%)	0.26x10^5 (76%)	0.41x10^5 (52%)	0.08x10^5 (64%)	3.62x10^5 (81%)	2.54x10^5	6.16x10^5
**Asia**	7.23x10^5 (80%)	1.30x10^5 (60%)	2.14x10^5 (44%)	0.68x10^5 (71%)	11.35x10^5 (71%)	9.73x10^5	21.1x10^5
**Europe**	1.3x10^5 (77%)	0.19x10^5 (60%)	0.34x10^5 (43%)	0.18x10^5 (54%)	2.04x10^5 (68%)	2.78x10^5	4.82x10^5
**Oceania**	1.6x10^5 (87%)	0.14x10^5 (83%)	0.06x10^3 (63%)	0.02x10^5 (79%)	1.82x10^5 (86%)	0.28x10^5	2.10x10^5
**North America**	2.9x10^5 (85%)	0.26x10^5 (72%)	0.79x10^5 (53%)	0.36x105 (75%)	4.34x10^5 (78%)	13.8x10^5	18.1x10^5
**South America**	5.7x10^5 (91%)	0.56x10^5 (83%)	0.98x10^5 (52%)	0.15x10^5 (72%)	7.36x10^5 (85%)	1.55x10^5	8.91x10^5
**Total**	22.7x10^5 (85%)	2.70x10^5 (69%)	4.72x10^5 (48%)	1.46x10^5 (70%)	30.5x10^5 (77%)	30.6x10^5	61.2x10^5

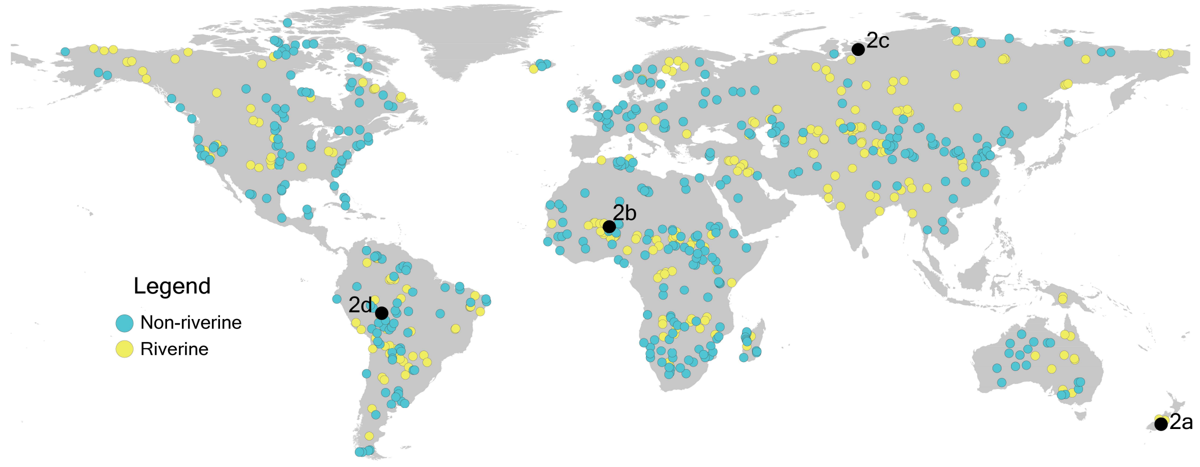

Supplementary Figure 1 Global Channel Belt Training Locations – Global distribution of riverine and non-riverine locations used to train and validate the machine learning algorithm. Overlain numbers refer to the examples shown in Supplementary Figure 2. Basemap of continents are dervied from the publicly available Global Shoreline Vector (GSV)[1,2] dataset.

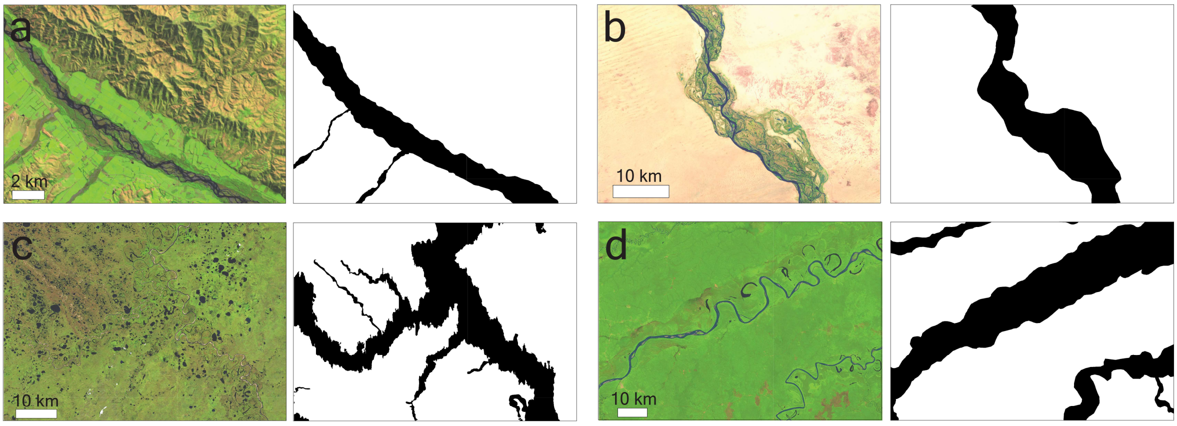

Supplementary Figure 2 Global Channel Belt Training Examples – Typical examples of binary training images used in the Global Channel Belt (GCB) machine learning algorithm showing the original 2020 Landsat 8 image and the mask highlighting the channel belt extent. a) Multi-threaded Waitaki River, New Zealand b) Multi-threaded Niger River, Mali c) Single-threaded unnamed river, Gydan Peninsula, Russia d) Single-threaded Rio Madre De Dios, Bolivia. Landsat-8 images courtesy of the U.S. Geological Survey.

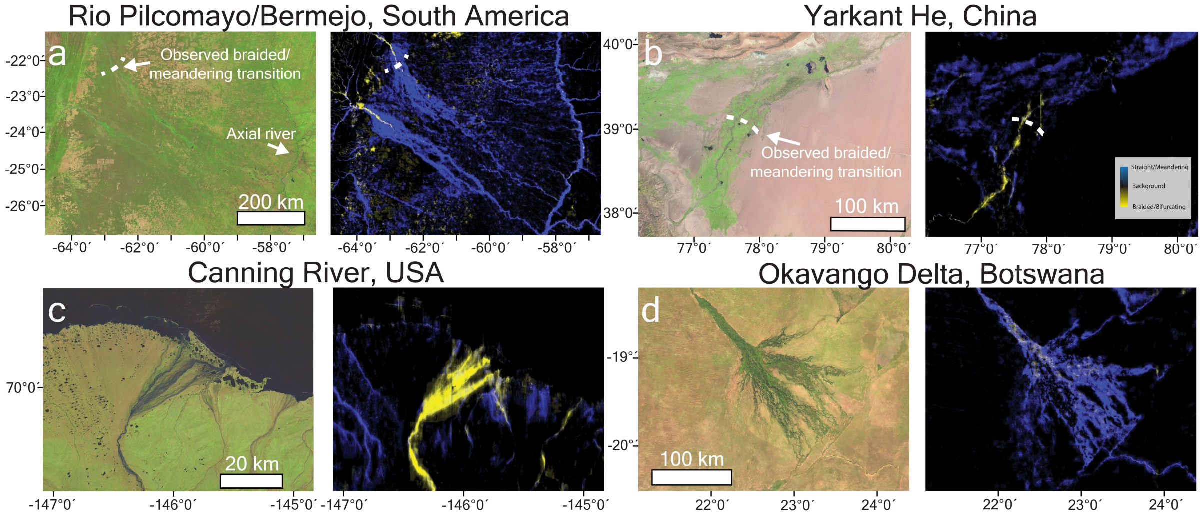

Supplementary Figure 3 Example Global Channel Belt Extent Predictions – Examples of the GCB prediction for different river systems covering a range of climates and tectonic settings versus the original 2020 Landsat 8 imagery. The observed braided to meandering transition in a and b are based on descriptions previously reported by Hartley et al.[3] and Davidson et al.[4]. Landsat-8 images courtesy of the U.S. Geological Survey.

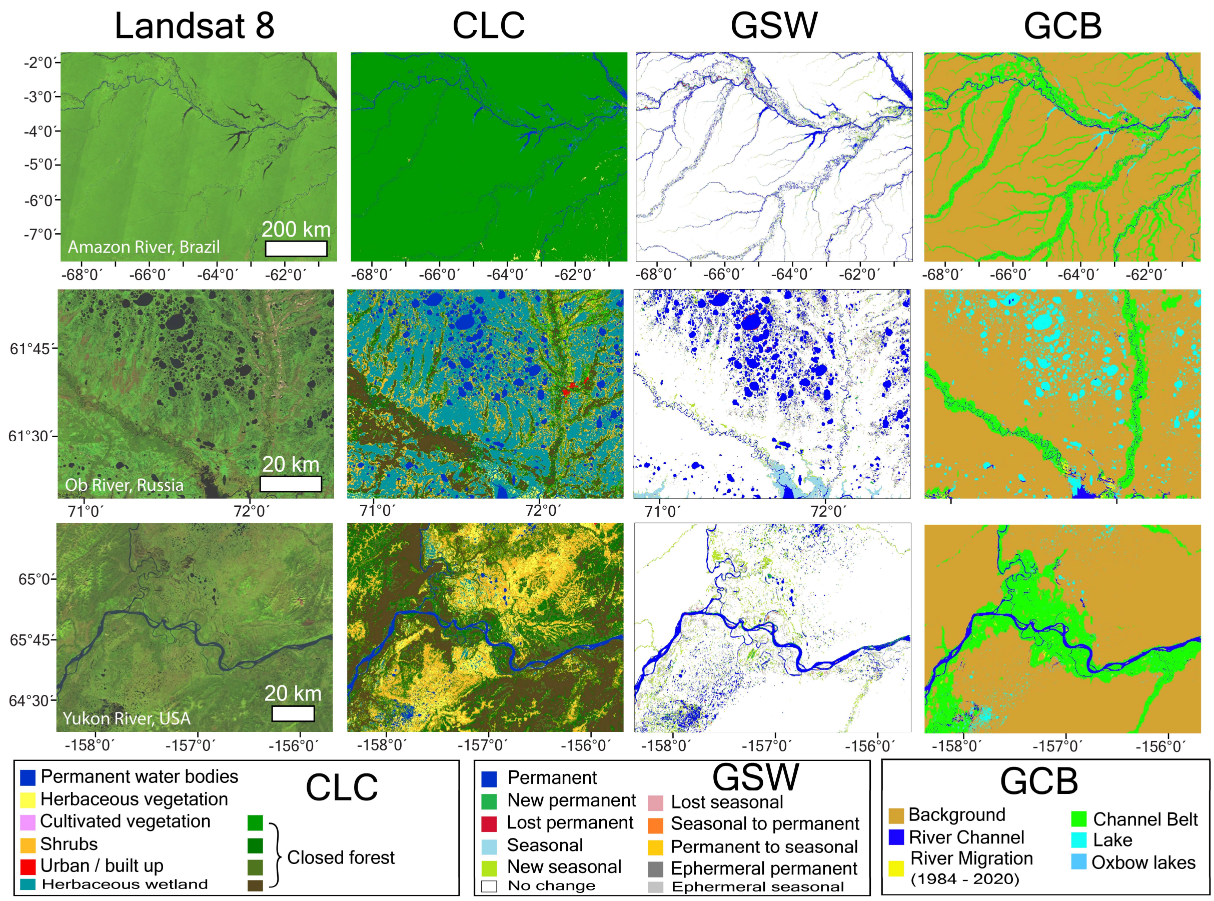

Supplementary Figure 4 Global Riverine and Lacustrine Environment Examples – Comparison of different publicly available global land and water classifications (Copernicus Land Cover (CLC) dataset[5], Global Surface Water (GSW) dataset[6] and the Global Channel Belt (GCB) dataset). Landsat-8 images courtesy of the U.S. Geological Survey.

References:

1. Sayre, R. *et al.* A new 30 meter resolution global shoreline vector and associated global islands database for the development of standardized ecological coastal units. *J. Oper. Oceanogr.* **12**, S47–S56 (2019).

2. Sayre, R., 2023, Global Islands: U.S. Geological Survey data release, https://doi.org/10.5066/P91ZCSGM

3. Hartley, A. J., Weissmann, G. S., Nichols, G. J. & Warwick, G. L. Large Distributive Fluvial Systems: Characteristics, Distribution, and Controls on Development. *J. Sediment. Res.* **80**, 167–183 (2010).

4. Davidson, S. K., Hartley, A. J., Weissmann, G. S., Nichols, G. J. & Scuderi, L. A. Geomorphic elements on modern distributive fluvial systems. *Geomorphology* **180–181**, 82–95 (2013).

5. Buchhorn, M., Smets, B., Bertels, L., Roo, B. de, Lesiv, M., Tsendbazar, N.-E., Herold, M., Fritz, S. (2020). Copernicus Global Land Service: Land Cover 100m: collection 3: epoch 2019: Globe. doi.org/10.5281/zenodo.3939050

6. Pekel, J. F., Cottam, A., Gorelick, N. & Belward, A. S. High-resolution mapping of global surface water and its long-term changes. *Nature* **540**, 418–422 (2016).
